# Soybean-Nodulating Strains With Low Intrinsic Competitiveness for Nodulation, Good Symbiotic Performance, and Stress-Tolerance Isolated From Soybean-Cropped Soils in Argentina

**DOI:** 10.3389/fmicb.2019.01061

**Published:** 2019-05-14

**Authors:** Esteban T. Iturralde, Julieta M. Covelli, Florencia Alvarez, Julieta Pérez-Giménez, Cesar Arrese-Igor, Aníbal R. Lodeiro

**Affiliations:** ^1^Facultad de Ciencias Exactas, Instituto de Biotecnología y Biología Molecular (IBBM), UNLP y CCT La Plata-CONICET, La Plata, Argentina; ^2^Institute for Multidisciplinary Research in Applied Biology (IMAB), Universidad Pública de Navarra, Pamplona, Spain

**Keywords:** *Bradyrhizobium*, allochthonous population, N_2_-fixation, inoculant, nodulation

## Abstract

Soybean is the most important oilseed in the world, cropped in 120–130 million hectares each year. The three most important soybean producers are Argentina, Brazil, and United States, where soybean crops are routinely inoculated with symbiotic N_2_-fixing *Bradyrhizobium* spp. This extended inoculation gave rise to soybean-nodulating allochthonous populations (SNAPs) that compete against new inoculant for nodulation, thus impairing yield responses. Competitiveness depends on intrinsic factors contributed by genotype, extrinsic ones determined by growth and environmental conditions, and strain persistence in the soil. To assess these factors in Argentinean SNAPs, we studied 58 isolates from five sites of the main soybean cropping area. BOX-A1R DNA fingerprint distributed these isolates in 10 clades that paralleled the pHs of their original soils. By contrast, reference *Bradyrhizobium* spp. strains, including those used as soybean-inoculants, were confined to a single clade. More detailed characterization of a subset of 11 SNAP-isolates revealed that five were *Bradyrhizobium japonicum*, two *Bradyrhizobium elkanii*, two *Rhizobium radiobacter* (formerly *Agrobacterium tumefaciens*), one *Bradyrhizobium diazoefficiens*, and one *Paenibacillus glycanilyticus*-which did not nodulate when inoculated alone, and therefore was excluded from further characterization. The remaining subset of 10 SNAP-isolates was used for deeper characterization. All SNAP-isolates were aluminum- and heat-tolerant, and most of them were glyphosate-tolerant. Meanwhile, inoculant strains tested were sensitive to aluminum and glyphosate. In addition, all SNAP-isolates were motile to different degrees. Only three SNAP-isolates were deficient for N_2_-fixation, and none was intrinsically more competitive than the inoculant strain. These results are in contrast to the general belief that rhizobia from soil populations evolved as intrinsically more competitive for nodulation and less N_2_-fixing effective than inoculants strains. Shoot:root ratios, both as dry biomass and as total N, were highly correlated with leaf ureide contents, and therefore may be easy indicators of N_2_-fixing performance, suggesting that highly effective N_2_-fixing and well-adapted strains may be readily selected from SNAPs. In addition, intrinsic competitiveness of the inoculants strains seems already optimized against SNAP strains, and therefore our efforts to improve nodules occupation by inoculated strains should focus on the optimization of extrinsic competitiveness factors, such as inoculant formulation and inoculation technology.

## Introduction

Soybean, which is one of the most important crops in the world, originated in China, where it was domesticated around 5,000 years ago. Accordingly, the highest diversity of soybean-nodulating rhizobia may be found in the North China Plain (Zhang et al., 2011), suggesting that this may be also the center of origin of this soil bacterial group. Subsequently, the soybean crop was extended to all over the world, and currently its major production area is in the Americas, mostly in Argentina, Brazil, and United States. In Argentina-at the China’s antipodes-there are mentions of soybean plantings as early as 1862, and the commercialization of an inoculant for soybean containing “*Bacterium radicicola soja*” is documented already 1932. However, by that time the crop was marginal and had to wait until 1962 to reach 10,000 ha, being exported for the first time in that year (Martínez-Alvarez, 2012). Afterwards, thanks to the sustained research efforts since the 1970s, and the release of glyphosate-resistant transgenic soybean cultivars in 1995, Argentina became the third soybean producer in the world, with around 20 million ha currently dedicated to this crop, and a mean yield of *ca*. 3 ton⋅ha^-1^. Because Argentina is not a center of origin for soybean, its soils should have been devoid of soybean-nodulating rhizobia before the introduction of this crop and its inoculants. In agreement to this notion, a survey of more than 700 field assays carried out by the National Institute of Agricultural Research (INTA) between 2000 and 2007 showed increases of at least 50% in grain yield in response to inoculation in soils with no previous history of soybean and without nutrient or water limitations. Meanwhile, in soils that included soybean in previous rotations, the response to inoculation dropped to averages of 6–10% increase in grain yield (Piccinetti et al., 2013).

This difference in response may be due to the incorporation of inoculants mainly based on *Bradyrhizobium diazoefficiens*, *Bradyrhizobium elkanii*, and *Bradyrhizobium japonicum* to the soybean-cropped soils, which soon established as allochthonous components of the local soil microbiota giving rise to a soybean-nodulating allochthonous population (SNAP). Genetic changes accumulated then in SNAPs, leading them to diverge from the original inoculants and to fit the Argentinean soils (Melchiorre et al., 2011). As consequence, SNAPs now compete with the high-quality inoculated strains for the occupation of soybean nodules (López-García et al., 2009), which may explain the poor responses often obtained regardless of soil quality and climatic conditions (Piccinetti et al., 2013). In this way, the problem of competition for nodulation looks as a main bottleneck for the success of soybean inoculation in Argentina.

Earlier studies in the United States Midwest, where *Bradyrhizobium* spp. were also introduced with inoculation, indicated that the serogroup 123 dominates the SNAPs, and it is highly competitive for nodulation of commercial soybean cultivars when it is co-inoculated with strains from other serogroups (Damirgi et al., 1967; Ellis et al., 1984; Moawad et al., 1984; Keyser and Cregan, 1987). These observations lead to the paradigm that SNAPs evolve to become highly competitive. So redoubtable the competition for nodulation is, that only a few hundreds of rhizobia in the soil population are enough to block any yield response of an inoculant carrying more than a 1,000-fold excess of active rhizobia (Thies et al., 1991a,b). Therefore, the question arose as how to improve the high-quality inoculant strains to render them also highly competitive (Triplett and Sadowsky, 1992; Stephens and Rask, 2000; Pérez-Giménez et al., 2011; Geetha and Joshi, 2013; Checcucci et al., 2017; Onishchuk et al., 2017).

At inception of the symbiotic relationship, the rhizosphere is colonized by rhizobia either from the soil population or from the inoculant. During this process, the rhizospheric rhizobial population may reach millions of cells, which will give rise to only a few hundreds of nodules at most in the mature root. Hence, competition among the different rhizobial genotypes present in the rhizosphere to occupy the root nodules should be harsh. Several steps are required for rhizospheric rhizobia to succeed in occupying a nodule, namely: adhesion to, and colonization of the root surface, induction of root hairs curling, infection of the root hairs through infection threads, exchange of chemical signals with the root host to trigger nodule development, colonization of the nodule, and differentiation into functional N_2_-fixing bacteroids (Murray, 2011; Oldroyd et al., 2011; Suzaki and Kawaguchi, 2014; Wheatley and Poole, 2018). Each step has a barrier to the progress toward the next and so, competition for nodulation may be affected at any of these barriers. Accordingly, bacterial genes acting in transitions between steps or in metabolic pathways involved in these processes, were identified as determining rhizobia competitiveness (Triplett and Barta, 1987; Triplett, 1988, 1990; Soto et al., 1993; Jiménez-Zurdo et al., 1995; van Dillewijn et al., 2001; Loh et al., 2002; Okazaki et al., 2003; Miller et al., 2007; Pobigaylo et al., 2008; Mongiardini et al., 2009; Patankar and González, 2009; Sánchez et al., 2009; Quelas et al., 2010, 2013, 2016b; Althabegoiti et al., 2011; Ding et al., 2012; Frederix et al., 2014; Geddes et al., 2014; Liu et al., 2017).

Other studies demonstrated that the competitiveness of a given genotype may be modified by manipulating external factors. The physiological state of the rhizobia at the time of inoculation seems relevant, as exponentially growing rhizobia are more infective and competitive than isogenic stationary-phase cultures (López-García et al., 2001, 2002). The composition of the culture medium, and in particular, its carbon:nitrogen ratio may condition the competitiveness (Fernandez-Flouret and Cleyet-Marel, 1987; López-García et al., 2001). Pretreatment of rhizobia with stimulating substances such as flavonoids and lectins also improves their competitiveness (Lodeiro et al., 2000b; Maj et al., 2010). Moreover, the soil environment has different influences on the competitiveness of certain genotypes. The acid soil pH favors competitiveness of slow-growing soybean-nodulating rhizobia over the fast-growing ones, while the alkaline soil pH does the contrary (Yang et al., 2001). Motility of inoculated rhizobia in soils at field-capacity is generally scarce, and therefore the distribution of the rhizobia in the soil profile is important as it facilitates that the growing roots enter in contact with the static rhizobia (López-García et al., 2002, 2009). Therefore, inoculant competitiveness may be improved not only genetically, but also through the control of external factors, such as bacterial growth and storage, composition of the culture media, addition of stimulating substances, and in-furrow inoculation instead of seed inoculation.

It is believed that the more competitive strains also may persist for longer periods in the soil, thus dominating the soil population, and enhancing their chances to occupy most of the nodules in a next crop cycle (Daubech et al., 2017; Onishchuk et al., 2017). Moreover, even in fields under soybean monoculture the free-living state out of the soybean nodules occupies a considerable portion of the SNAP life-time. Therefore, the adaptations to saprophytic life in the soil might evolve beyond the ability to nodulate in the local conditions (Hollowell et al., 2016). By contrast, inoculated rhizobia are often adapted to laboratory growth conditions, and may lack adaptations to the specific stress conditions that will have to face once released in a given soil. Since competitive nodulators need not to be excellent N_2_-fixers, there is no apparent reason for natural selection of a high N_2_-fixation performance in the soil environment. As a result of several years of adaptation the SNAP might be enriched in genotypes of high competitiveness for nodulation and high adaptation to persist in the local environment, while diverging in its nitrogen fixation efficiency, allowing the appearance of “cheaters” that do not return the expected fixed nitrogen to the host plant (Sachs et al., 2010; Schumpp and Deakin, 2010).

Therefore, inoculants counteraction by the SNAP may be due to its intrinsic competitiveness (i.e., the part genetically determined), its extrinsic competitiveness (i.e., the part determined by external factors acting differentially on the inoculant or the SNAP, or acting differentially on different genotypes), its adaptation and persistence (which determine the effective number of SNAP active competitors), and its variable N_2_-fixing efficacy. Consequently, better knowledge of the SNAP seems necessary to improve inoculation strategy and performance. Despite this, the SNAPs intrinsic competitiveness was seldom evaluated, and in particular, few studies were aimed at characterizing the SNAPs in Argentina (Melchiorre et al., 2011; López et al., 2013). We believe that it is important to confirm whether the SNAP members actually possess high intrinsic competitiveness, or if its ability to occupy most of the nodules of soybean crops is due to extrinsic competitiveness and persistence. Assessing these alternatives might suggest whether it is worth doing the effort of genetically breeding strains with superior intrinsic competitiveness, or if would be more rewarding to improve inoculant application technologies. To gain more information about the properties of Argentinean SNAPs, here we isolated soybean-nodulating rhizobia from five locations in the main soybean-producing area of Argentina. Then, we characterized the isolates both genotypically and phenotypically, and with a subgroup of isolates we inquired on their symbiotic performance and intrinsic competitiveness for nodulation.

## Materials and Methods

### Isolation of Soybean-Nodulating Bacteria From SNAPs

The isolates used in this study were obtained from five sites in the main soybean cropping area of Argentina. The sites were: the experimental station of INTA-Castelar (CAS), Province of Buenos Aires (34°36′15′′S; 58°40′27′′W); the Kilgruman farm at Cavanagh (CAV), Province of Santa Fe (33°27′42′′S; 62°17′9′′W); the experimental field of INTA-Concepción del Uruguay (CUR) at Villa Mantero, Province of Entre Ríos (32°23′17′′S; 58°45′20′′W); the experimental field of INTA-Nueve de Julio (NUJ) at French, Province of Buenos Aires (35°30′54′′S; 61°00′04′′W); and the experimental field of INTA-San Antonio de Areco (SAA) at the farm La Fe, Province of Buenos Aires (34°15′00′′S; 59°28′00′′W). The following soil properties were determined at CIRN-INTA (Argentina): soil texture by sedimentation volume, organic carbon according to Walkley and Black (1934), organic matter from organic carbon using Van Benmelen factor, organic nitrogen by Kjeldahl method, available phosphorous according to Olsen et al. (1954), and pH (in water) in 1:2.5 suspension (Table 1). All fields had at least 5 years of previous history of soybean crops, and were under experimentation conducted by INTA to evaluate different soybean inoculants. The samplings were performed with permission from on-site staff from INTA.

**Table 1:** Main characteristics of the soils from where the soybean-nodulating isolates were obtained.

Characteristic	Site				
	CAS	CAV	CUR	NUJ	SAA
Texture (% clay : lime : sand)	25.8:52.6:21.6	19.5:49.7:30.8	40.2:57.5:2.3.	14.7:29.2:56.1	31.1:56.5:12.4
Structure	Sub-angular blocks	Sub-angular blocks	Sub-angular blocks	Sub-angular blocks	Sub-angular blocks
Organic matter (%)	4.60	2.38	4.31	2.90	3.28
Organic nitrogen (%)	0.26	0.14	0.18	0.13	0.18
C/N ratio	10.3	9.8	13.9	10.2	10.6
Available phosphorous (ppm)	5.8	10.7	5.8	16.4	18.6
pH (in H_2_0)	5.7	5.6	6.4	6.2	4.8

*CAS, Castelar; CAV, Cavanagh; CUR, Concepción del Uruguay; NUJ, Nueve de Julio; SAA, San Antonio de Areco*.

We obtained the samples from non-inoculated control plots, in two ways. On the one hand, we selected at least 5 plants from the middle of the plots and cut their roots, which we stored refrigerated. On the other hand, 7 samples of surface soil were collected at the vertices of a zigzag from each plot, then mixed, and stored in plastic bags. All samples were transported to the laboratory as soon as possible. Once in the laboratory, we selected the 1–2 uppermost nodules of each plant, which were surface-sterilized, crushed, and their contents cultured in yeast-extract-mannitol with 1.5% agar (YMA) as previously described (Althabegoiti et al., 2011) to obtain the “nodule” (N) samples. From the soil samples, 100 g soil were suspended in 900 ml of modified Fåhraeus N-free plant nutrient solution (MFS) (Lodeiro et al., 2000a), agitated vigorously, diluted, and inoculated at 3 ml plant^-1^ onto four surface-sterilized soybean seedlings, which were previously planted in vermiculite pots watered with MFS as described (López-García et al., 2001). In addition, uninoculated controls were maintained to detect possible cross-contamination. After 25 days in the greenhouse and absence of contamination being ensured, we excised the 1–2 uppermost nodules from each plant, surface-sterilized them, crushed them, and streaked their contents in YMA as before, to obtain the “soil” (S) samples. After culturing the N and S samples to get bacterial growth, the bacterial colonies obtained were purified by repeated streaking on YMA medium. We suspended the bacteria from isolated colonies in MFS, and inoculated soybean plants as described above. Three weeks later, we re-isolated the bacteria from the nodules, isolated the colonies, and stored these isolates at -80°C as stocks in liquid YM broth (YMB) supplemented with 20% glycerol (v/v).

We denominated our SNAP-isolates with the three letters that indicate the site of origin, followed by a forward slash and the letter that indicates whether the isolate comes from a field nodule or from a soil sample, and finally a number separated by a middle script that identifies the isolate. For instance, the denomination “CUR/S-21” names the isolate #21, which was obtained from a soil sample in Concepción del Uruguay.

In addition to these isolates, we used as reference strains *B. diazoefficiens* USDA 110^T^ (obtained from Deane Weber, ARS-USDA, United States), *B. japonicum* E109 (widely used in Argentinean inoculants, obtained from Alejandro Perticari, IMYZA-INTA, Argentina), *B. diazoefficiens* USDA 122, *B. elkanii* USDA 76^T^, *B. elkanii* USDA 94, *B. japonicum* USDA 6^T^ (all obtained from Esperanza Martínez-Romero, Centro de Ciencias Genómicas, UNAM, México), *B. diazoefficiens* SEMIA 5080, and *B. japonicum* SEMIA 5079 (both widely used in Brazilian inoculants and to some extent in Argentina, obtained from Mariangela Hungria, EMBRAPA, Brazil). All strains were maintained as glycerol stocks as described for the field isolates.

For routine use, samples from glycerol stocks were grown in YMA and maintained at 4°C for no more than 3 months. Except when indicated, the bacteria from YMA stocks were grown for experimental use in YMB with rotary shaking at 180 rev⋅min^-1^ or in YMA, in both cases at 28°C. The biomass and the viable cell numbers were estimated in liquid cultures by the optical density (500 nm) or the colony-forming units (CFU) counts, respectively.

### DNA Purification

We carried out DNA purification as described previously (Walsh et al., 1991). Briefly, we suspended single bacterial colonies in 300 μl 1 M NaCl, vortexed, and incubated these suspensions at 4°C overnight; then we centrifuged the samples at maximum speed for 4 min in a microcentrifuge and washed the pellet twice with 300 μl bidistilled water. We added 150 μl Chelex-100 (Bio-Rad) 6% (w/v) resin while stirring. We incubated the suspension at 56°C 20 min, vortexed, and incubated again 8 min at 99°C. Finally, we vortexed and stored the DNA bound to the resin at 4°C.

### PCR

For DNA-fingerprint we used the primer BOX-A1R (Koeuth et al., 1995). The reaction mixture (20 μl final volume) contained Tris-HCl 10X buffer, 2.5 mM MgCl_2_, 200 μM dNTPs, 2 units Taq DNA polymerase (all reactants from Invitrogen, Buenos Aires, Argentina), 20 μM BOX-A1R primer, which was synthesized by Operon Co. (United States), and 5 μl template DNA (∼20 ng) obtained as described in subsection “DNA Purification.” The cyclic conditions were: initial denaturation: 95°C 5 min, 35 cycles at 94°C 1 min, 52°C 1 min, 65°C 8 min, and final elongation: 68°C 16 min. For 16S rRNA gene, *atpD*, *glnII*, and *recA* fragments we employed the primers and PCR conditions described by Delamuta et al. (2013). For *nodC* and *nifH* amplification we used the conditions and primers described by Laguerre et al. (2001).

### DNA-Fingerprint

We separated the BOX-A1R PCR products in agarose (2% w/v) gel electrophoresis at 70 volt during 5 h. We normalized the gel photographs with reference lanes consisting of molecular weight standards (DNA ladder plus 100 bp, Genbiotech, Buenos Aires, Argentina) and reaction products with DNA from *B. diazoefficiens* USDA 110. Then we analyzed each SNAP lane for the presence or absence of bands with the GelCompare II 4.0 software (Applied Maths, Kortrijk, Belgium). We optimized the band pattern with a 1.5% tolerance. We obtained the cladogram with the unweighted pair-group method, with arithmetic mean (UPGMA) (Sneath and Sokal, 1973) and evaluated the distances among branches with the Jaccard coefficient (Jaccard, 1912).

### DNA Sequencing

DNA products were sequenced at Macrogen (Korea) with an ABI 3730xl DNA Sequencer. Then, we analyzed the 16S rRNA, *recA*, *atpD*, and *glnII* sequences obtained with Bioedit, version 7.2.5. We performed the MLSA analysis by considering only the complete aligned SNAP sequences and the type/reference strain sequences (size among parenthesis) retrieved from GenBank: 16S rDNA (1,347 bp), *recA* (293 bp), *atpD* (395 bp), *glnII* (442 bp), *nodC* (approximately 930 bp), and *nifH* (780 bp). All sequences obtained in this study or retrieved from GenBank were analyzed individually and concatenated using MEGA 7 (Molecular Evolutionary Genetics Analysis Version 7, Kumar et al., 2016).

### MALDI-TOF MS Analysis

We performed the identification and classification of rhizobia by Matrix-Assisted Laser Desorption/Ionization Time-Of-Flight Mass Spectrometry (MALDI-TOF MS) using an Ultraflex III MALDI-TOF/TOF mass spectrometer and the MALDI Biotyper 3.1 software (Bruker Daltonics, Bremen, Germany). We built a reference database with *Rhizobium* and *Bradyrhizobium* strains as previously reported by Ferreira et al. (2011) and Sánchez-Juanes et al. (2013), and included it in the extended MALDI Biotyper 3.1 library database. We prepared the samples according to manufacturers’s recommendation, either by picking a single colony (direct smear) or using 1 μl of protein-containing supernatant (ethanol/formic acid extraction mehtod) from *Bradyrhizobium* spp. and *Rhizobium* spp. strains cultured in peptone-salts-yeast extract (PSY) medium (Regensburger and Hennecke, 1983) supplemented with 0.1% arabinose or triptone-yeast extract (TY) medium (Smit et al., 1986), respectively. We spotted the samples onto the MALDI target and overlaid them with 1 μl of satured solution of α-cyano-4-hydroxycinnamic acid in organic solution (50% acetonitrile, 2.5% trifluoroacetic acid). We recorded the spectra by Flex Control 3.3 software (Bruker Daltonics) as described (Ferreira et al., 2011). We classified MALDI-TOF MS identifications using the score values described in Ferreira et al. (2011): 1.70–1.99: probable genus identification; 2.0–2.29: probable species identification; >2.3: highly probable species identification.

### Stress Tolerance

We evaluated the capacity of the isolates to grow at low pH and in the presence of aluminum according to Ayanaba et al. (1983). Briefly, we cultivated the bacteria in YMA supplemented with bromothymol blue 25 μg⋅l^-1^ pH = 4.8 in the presence or absence of 50 μM AlCl_3_ for 10 days at 28°C, and visualized the tolerance as the ability to form a bacterial mass on the agar surface in these conditions.

To evaluate the glyphosate tolerance we used the method described by Moorman et al. (1992). We cultured the bacteria in the aromatic aminoacid-free liquid MSR medium, with rotary shaking at 180 rev⋅min^-1^ and 28°C up to an approximate cell density of 10^8^ cells⋅ml^-1^, which constituted the starter culture. We diluted this culture to ca. 10^7^ cells⋅ml^-1^ in MSR with or without 2.5 mM glyphosate (Sigma-Aldrich, Buenos Aires, Argentina). We then grew the cultures at 28°C and 180 rev⋅min^-1^ for an additional 3 days and compared the final CFU⋅ml^-1^ achieved in the absence or in the presence of glyphosate. We quantified the glyphosate tolerance as the percentage of CFU⋅ml^-1^ in the medium with glyphosate with respect to the CFU⋅ml^-1^ in the medium without glyphosate.

We evaluated heat tolerance as the % CFU after submitting YMB bacterial cultures to a temperature climb from 28 to 40°C at a rate of 1°C every 15 min, followed by a temperature descend back to 28°C at the same rate.

### Motility Assays

To evaluate swimming motility, we inoculated the bacteria with a toothpick in semisolid Götz agar (0.3%) and observed the bacterial dispersion at 28°C as described (Althabegoiti et al., 2011).

### Plant Assays

We surface-sterilized and germinated the soybean seeds DonMario 4800, widely used for cropping in the sampled area, as previously described (López-García et al., 2001). Then, we planted one germinated seedling in each one of the required number of 600 ml Ta-Tay tuppers (Plásticos Ta-Tay SA Barcelona, Spain), which were previously filled with sterile vermiculite/perlite (2:1) and initially watered with 250 ml sterile MFS. Each tupper possessed a screw-cap with a 5-cm hole to allow the shoot passing through it. To inoculate the plants, we suspended single bacterial colonies from YMA stocks in 1 ml sterile MFS and deposited each 1 ml-suspension onto each seed. Control plants were inoculated with 1 ml of sterile MFS without bacteria. Then, we cultured the plants for 50 days in a plant growth chamber at 26°C/18°C day/night temperature, 60%/80% day/night relative humidity, and a photophase of 16 h. During this period, we watered the plants three times per week (once with sterile MFS and twice with sterile distilled water).

We assayed 8 plants inoculated with each of the bacterial isolates tested (N and S samples), plus 8 uninoculated plants as controls, and 8 plants inoculated with either *B. diazoefficiens* USDA 110 or *B. japonicum* E109 as reference strains. From each plant we separated the shoot, the root, and the nodules, and dried all the materials at 60°C up to weight constancy. After determining the corresponding dry weights and the number of nodules per plant, we determined the shoot and root total nitrogen contents by the method of Kjeldahl (Eastin, 1978). In parallel, we obtained fresh leaf samples from each plant to estimate their ureide contents.

### Ureide Determinations

To estimate the ureide contents in leaves, we homogenized 10-mg samples of fresh leaf tissue with 1 ml 0.2 N NaOH during 30 min at 100°C. Then, we centrifuged the homogenates at 10,000 × *g* for 10 min in a microcentrifuge, and quantified the ureides concentrations in the clear supernatants as the phenylhydrazone of glyoxylate (Vogels and van der Drift, 1970).

### Competition for Nodulation

To distinguish the strains that occupied nodules in combined inoculations, we used *B. japonicum* LP 3018, a streptomycin-resistant/spectinomycin-resistant (Sm^r^/Sp^r^) derivative from *B. japonicum* E109 (López-García et al., 2009) as the reference strain. Since the physiological growth state strongly influences competitiveness, we intended to prepare the inoculant admixtures with bacteria in exponential growth phase. To this end, we suspended single bacterial colonies from YMA stocks in AG liquid medium (Sadowsky et al., 1987) and cultured these starters at 28°C with rotary shaking at 180 rev⋅min^-1^ up to saturation. Then, we diluted the starters 1:100 in fresh AG and continued their culturing during the time required to reach the mid-exponential phase. At this point, we diluted these vigorously growing cultures 1:50 in fresh AG and cultured them in the same conditions again up to mid-exponential phase. With such cultures we prepared 1:1 bacterial admixtures containing the assayed strain and the reference *B. japonicum* LP 3018 strain. To ensure the presence of 1⋅10^6^ cells of each strain in the mixed inoculants, we counted the total cell numbers of each culture with a Neubauer chamber under the microscope, and diluted the cultures accordingly before mixing. In parallel, we plated serial dilutions of the samples before mixing in YMA to count the CFU inoculated. The direct bacterial counts and the CFU counts closely matched so that we expressed the isolate:LP3018 ratio with the mean values from these two alternative cell counts.

We watered the tuppers with 250 ml of each of these admixtures prepared in MFS, added one seedling per tupper, and grew them in the above-described conditions. We used 15 plants per admixture, and included groups of 5 plants inoculated with each strain alone, plus a group of 5 uninoculated plants. Thirty days after inoculation we removed the nodules, then surface-sterilized them, and obtained and cultured the nodule-occupying strains as described (Althabegoiti et al., 2011). To distinguish the reference strain LP 3018 from the isolates, we inoculated each nodule extract with a sterile toothpick in two replica YMA plates prepared with or without the addition of 400 mg⋅ml^-1^ Sm plus 200 mg⋅ml^-1^ Sp. In this way, the spots that presented growth with the antibiotics indicated the presence of LP 3018 into the original nodule, while the spots sensitive to antibiotics corresponded to nodule extracts in which the LP 3018 reference strain was absent (for details, see Althabegoiti et al., 2011). We considered valid those assays in which 100% of the nodules from plants inoculated with single strains were occupied by the corresponding strains, and at the same time no nodules were formed in the uninoculated controls.

### Statistical Analyses

For the symbiotic parameters we employed the analysis of variance (ANOVA) followed by Tukey test to identify significant mean differences with a threshold of *p* < 0.05. We calculated the correlations between the different symbiotic parameters using the coordinate points obtained from each pair of parameters from averages of the same isolate, and calculating the ratio of the covariance to the product of the standard deviations extended to all the isolates. We then estimated the significance of the *r* correlation coefficient obtained in this way by comparing the calculated and tabulated Student’s *t*-values.

The distribution of isolates among clades and the distribution of nodule occupancies in the competition for nodulation experiments were assessed with a non-parametric χ^2^ test. Since in the competition for nodulation experiments we could not distinguish the nodules simultaneously occupied by an isolate and the LP 3018 reference strain (double occupation) from those occupied by only LP 3018 because both kinds of nodules released antibiotic-resistant bacteria, we assumed a 10% nodule double occupation, leading to a null hypothesis of 55% antibiotic-resistant:45% antibiotic-sensitive nodules (Althabegoiti et al., 2011).

To evaluate the similarity among the DNA-fingerprint genotypes, we generated similarity coefficients by the band-based method of Jaccard (1912) by using fuzzy logic and area-sensitive options. We calculated the Jaccard similarity coefficient for each pair of fingerprints by dividing the number of bands that occurred in both fingerprints by the total number of bands (common and unique) in both fingerprints. The fuzzy logic option allowed band matching values to gradually decrease with the distance between bands, and the area-sensitive option took into account differences in area between matching bands.

For analysis of DNA sequence data, we selected the DNA evolution model using jModelTest (Posada, 2008). Furthermore, we used the General Time Reversible model (GTR) Gamma distributed with Invariant sited (G+I) for 16S rRNA and concatenated genes. We grouped the sequences by maximum likelihood and evaluated the support for tree nodes by bootstrap analyses with 1,000 samplings (Felsenstein, 1985; Hedges, 1992).

### Accession Numbers

The gene sequences of 16S rRNA *recA*, *atpD*, and *glnII* were deposited in GeneBank, and the accession numbers assigned are listed in Supplementary Table S1.

## Results

### Genotypic Classification of the SNAP-Isolates

The SNAP-isolates employed in this study were obtained either from field nodules (N isolates) or from soil extracts (S isolates) from five representative sites of the most important soybean-producing area of Argentina (Table 1). We generated BOX-A1R DNA-fingerprints (Koeuth et al., 1995) with at least three biological replicas of each SNAP-isolate for their classification. During this procedure, we obtained non-reproducible DNA-fingerprint profiles among the replicas of the isolates CAV/S-14, CUR/S-25, and NUJ/N-44. The discrepancy in CAV/S-14 DNA-fingerprint profiles was of only one band, which was present in some replicas and absent in others, while the instability of CUR/S-25 and NUJ/N-44 was more pronounced. In addition, each of the last two isolates developed two types of colonies in the petri dishes, despite the previous colony purification and passage through nodules. However, even when we inoculated plants with one of such isolated colonies, the two colony morphologies reappeared when the bacteria were recovered again from the nodules. To strengthen the isolation procedure of CAV/S-14, CUR/S-25, and NUJ/N-44, we took single colonies, suspended them in buffer containing sodium deoxycholate and vortexed the suspensions at maximum speed for 5 min. Only after this procedure we could obtain pure cultures containing only one colony morphology that was recovered as unique after passage through nodules-except one that did not nodulate. We then renamed the soybean-nodulating isolates separated from these strong associations as CAV/S-14-1, CAV/S-14-2, CUR/S-25-2, NUJ/N-44-1, and NUJ/N-44-2, and the non-nodulating isolate as CUR/S-25-1.

**Figure 1:**
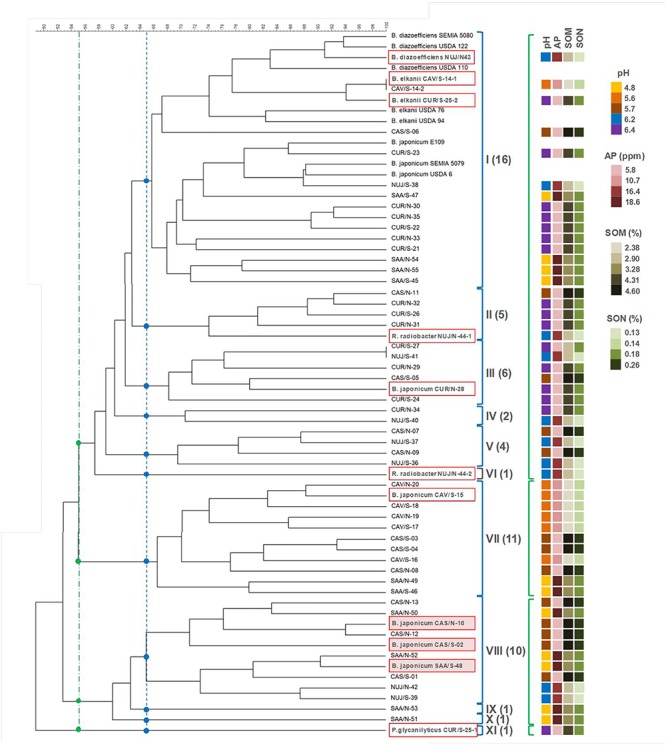
DNA-fingerprint of the SNAP-isolates and reference strains obtained with BOX-A1R primer. Clades were defined at 65% similarity (blue lines, dots, and brackets) or 55% similarity (green lines, dots, and brackets). The numbers of SNAP-isolates included in clades I–XI (at 65% similarity) are indicated in parenthesis. Boxed isolates are those picked for further species identification and characterization. Shadowed boxes indicate isolates that were equally competitive as the reference strain *B. japonicum* E109. Relevant soil properties of the soil of origin of each SNAP isolate are shown as colored squares on the right, according to the color scale for the values of soil properties detailed in Table 1. AP, available phosphorous; SOM, soil organic matter; SON, soil organic nitrogen.

We then classified the SNAP-isolates in a cladogram keeping the non-nodulating isolate CUR/S-25-1, which was identified as *Paenibacillus glycanilyticus* (see subsection “Assignment of Taxonomic Position”) as the outgroup (Figure 1). With a cutoff of 65% similarity (Chibeba et al., 2017) we grouped the SNAP-isolates in 10 clades plus the outgroup. Only three of the clades had more than nine SNAP-isolates each, while the remaining seven clades were smaller. By contrast, all the collection strains were closely grouped in clade I. Moreover, the *B. diazoefficiens* and *B. elkanii* collection strains were associated in a branch at 74% similarity, while the *B. japonicum* collection strains were associated in another, nearby branch with 79% similarity (Figure 1). A total of eight SNAP-isolates (including CAV/S-14-1 and CAV/S-14-2) were associated to the collection strains in these two branches. Clade I also included another nine SNAP-isolates, while the remaining 42 SNAP-isolates were distributed in the other 10 clades.

To see whether the distribution of the SNAP-isolates among the clades responds to the site of isolation or to the ability to nodulate in the local conditions, we performed a non-parametric χ^2^ test, selecting only those clades bearing at least 10 SNAP-isolates (clades I, VII, and VIII) to allow the random presence of at least one SNAP isolate from CAV, the least represented site (13.8% of total SNAP-isolates). Our null hypothesis was a random distribution of SNAP-isolates into each clade. Therefore, the expected values were obtained by multiplying the proportions of SNAP-isolates (discriminated as per site of isolation: CAS: 13/58; CAV: 8/58; CUR: 16/58; NUJ: 10/58; SAA: 11/58, or per method of isolation: N: 28/58; S: 30/58) times the total number of SNAP-isolates of each clade. This analysis indicated that the provenance of the samples was not randomly distributed in clade VII, with CAV isolates overrepresented (χ^2^ = 18.295; significant with *p* < 0.01). In addition, CUR isolates-the most abundant source-were absent in both clades VII and VIII (Figure 1). Among soil properties evaluated in Table 1, soil pH was the only that seemingly matched genotypes distribution. By lowering the similarity cutoff to 55%, a clade embracing clades I–VI results, which contains 23 SNAP-isolates from soils with pH higher than 6.0 from a total of 34 SNAP-isolates (68%), while the remaining clusters contain 24 SNAP-isolates, being only two of them (8%) from soils with pH > 6.0 (Figure 1). Meanwhile, the N or S isolates were randomly distributed in all three clades (χ^2^ values < 1; non-significant). Similar conclusions were obtained when we set the similarity cutoff at 60%, which rendered three major clades with more than 10 SNAP-isolates, and three minor clades (not shown).

### Assignment of Taxonomic Position

To perform a deeper study of the SNAP-isolates with regard to their taxonomic position and their phenotypic and symbiotic properties, we picked a group of 12 SNAP-isolates, comprising those that resulted from separation of the two colony morphologies that were persistently observed in subsection “Genotypic Classification of the SNAP-Isolates,” as well as representatives of the three major clades (Figure 1).

**Figure 2:**
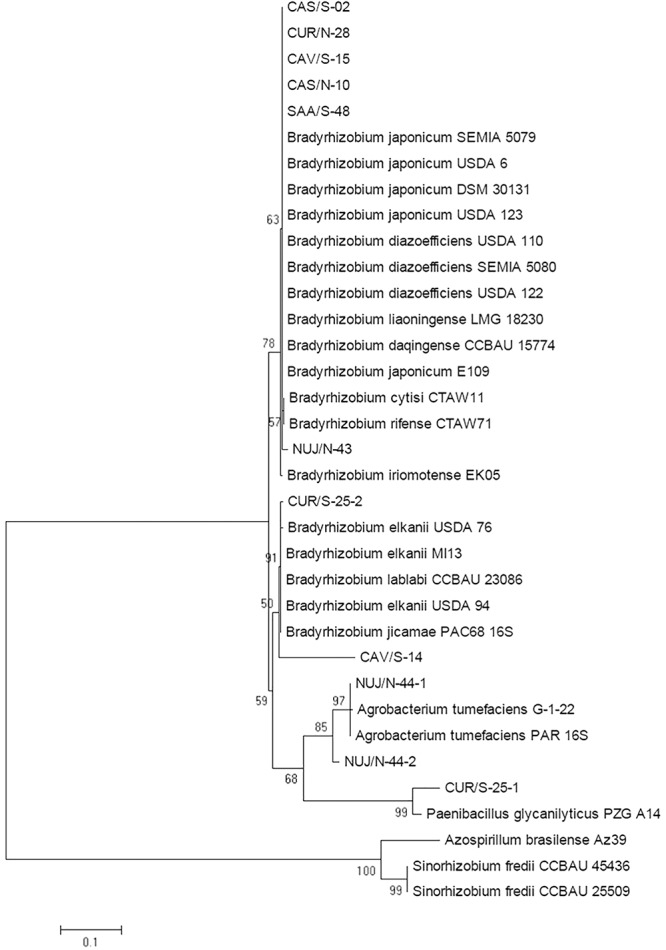
Maximum likelihood cladogram for the 16S rRNA sequences of picked SNAP-isolates and reference strains. Next to the nodes the percentage of replicate trees in which the associated taxa clustered together in the bootstrap test with 1,000 replicates are shown. Branch lengths in the tree are scaled in the same units as those of the evolutionary distances (scale bar) used to infer the phylogenetic tree.

As first approximation, we assigned the taxonomic positions of these SNAP-isolates by sequencing fragments of the 16S ribosomal RNA gene. Since the sequences from CAV/S-14-1 and CAV/S-14-2 were identical, the following results shall be reported for CAV/S-14-1, which will be referred to as CAV/S-14. The resultant tree allowed us to classify six SNAP-isolates as *Bradyrhizobium* spp., two as *B. elkanii*, two as *Rhizobium radiobacter* (formerly *Agrobacterium tumefaciens*), and the non-nodulating isolate CUR/S-25-1 as *P. glycanilyticus* (Figure 2). In agreement with their nodulating ability, we obtained *nodC* sequences from *R. radiobacter* NUJ/N-44-1 and NUJ/N-44-2 with 99.62 and 98.66% identity, respectively, with *R. radiobacter* MQ-110s, and *nifH* from NUJ/N-44-2 with 89.78% identity with *R. radiobacter* gx-178 (Supplementary Figure S1). We could not amplify *nifH* from NUJ/N-44-1. The cladograms constructed with these genes positioned both *R. radiobacter* isolates together with *R. radiobacter* (*A. tumefaciens*) MQ-110s and far away from *Bradyrhizobium* spp. (Supplementary Figure S2).

We corroborated these taxonomic positions by the alternative method of phenotypic identification based on MALDI-TOF MS using the Biotyper software, although with this method, NUJ/N-44-2 also matched with *Sinorhizobium* sp. (Table 2). In agreement with this classification, the growth rate of *R. radiobacter* and *P. glycanilyticus* isolates was fast (Table 2), giving rise to colonies in YMA after 2 days of incubation, by contrast to the *Bradyrhizobium* spp. isolates, which took 5 days to form colonies in YMA.

**Table 2:** Species determination and relevant properties of SNAP-isolates and reference strains (Ref.).

Isolate	Species (according to)		Tolerance against				Growth rate^f^ (h^-1^)	Swimming halo^g^ (mm)
	16S-RNA-sequencing	MALDI-TOF MS	Acidity^b^	Alumi-	Glyphosate^d^	Heat^e^		
	(% identity)	(Score)^a^		nium^c^				
CAS/N-10	*B. japonicum* (99)	*B. japonicum* (2.58)	Yes	Yes	23.5 ± 0.9	90.05 ± 3.63	0.06	23.0 ± 0.8
CAS/S-02	*B. japonicum* (99)	*B. japonicum* (2.40)	Yes	Yes	103.2 ± 21.2	92.78 ± 5.52	0.10	19.0 ± 1.4
CAV/S-14	*B. elkanii* (96)	*B. elkanii* (2.42)	Yes	Yes	79.0 ± 9.6	86.76 ± 3.60	0.08	40.0 ± 2.0
CAV/S-15	*B. japonicum* (99)	*B. japonicum* (2.33)	Yes	Yes	98.3 ± 22.7	109.18 ± 4.45	0.09	17.0 ± 1.0
CUR/N-28	*B. japonicum* (99)	*B. japonicum* (2.01)	Yes	Yes	75.00 16.1	92.62 ± 3.80	0.07	11.7 ± 1.5
CUR/S-25-1	*P. glycanilyticus* (99)	*Paenibacillus* (1.86)	Yes	Yes	ND^h^	91.25 ± 3.32	0.43	ND^h^
CUR/S-25-2	*B. elkanii* (98)	*B. elkanii* (2.46)	Yes	Yes	109.8 ± 21.3	92.41 ± 3.99	0.07	33.7 ± 1.1
NUJ/N-43	*B. diazoefficiens* (98)	*B. diazoefficiens* (2.22)	Yes	Yes	100.5 ± 21.2	98.17 ± 1.84	0.10	30.7 ± 2.1
NUJ/N-44-1	*R. radiobacter* (97)	*R. radiobacter* (2.27)	Yes	Yes	83.0 ± 12.8	94.32 ± 4.24	0.43	39.0 ± 1.0
NUJ/N-44-2	*R. radiobacter* (97)	*Sinorhizobium* (1.93)	Yes	Yes	45.1 ± 12.8	94.23 ± 2.05	0.18	32.3 ± 0.6
SAA/S-48	*B. japonicum* (99)	*B. japonicum* (2.37)	Yes	Yes	70.9 ± 14.8	83.43 4.08	0.06	22.3 ± 0.6
E109	*B. japonicum* (Ref.)	*B. japonicum* (Ref.)	Yes	No	38.6 ± 7.0	91.21 ± 3.68	0.10	24.4 ± 0.8
USDA 110	*B. diazoefficiens* (Ref.)	*B. diazoefficiens* (Ref.)	Yes	No	ND^h^	112.36 ± 6.75	0.09	21.6 ± 1.3

^a^*Score ranges: 1.70–1.99: probable genus identification; 2.0–2.29: probable species identification; >2.3: highly probable species identification*.

^b^*Presence/absence of bacterial growth in YMA at pH = 4.8*.

^c^*Presence/absence of bacterial growth in YMA at pH = 4.8 in the presence of 50 μM AlCl _3_*.

^d^*Percentage ± SD of surviving bacteria after 3 days in the presence of 2.5 mM glyphosate in MSR liquid medium*.

^e^*Percentage ± SD of surviving bacteria after incubation in a temperature scaling from 28 to 40°C and back to 28°C at 1°C⋅every 15 min*.

^f^*Obtained from the slope of the log_*10*_ optical density at 500 nm in YMB against time during exponential growth phase*.

^g^*Diameter ± SD of the swimming halo in Götz medium with 0.3% agar after 7 days (Bradyrhizobium spp.) or 4 days (R. radiobacter)*.

^h^*ND, not determined*.

**Figure 3:**
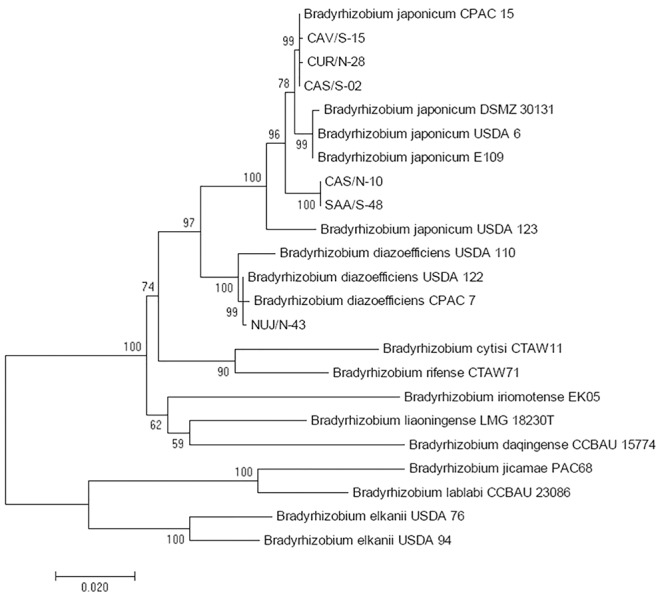
Maximum likelihood cladogram for the concatenated *atpD*, *glnII*, and *recA* fragment sequences to distinguish *B. diazoefficiens* from *B. japonicum* among picked SNAP-isolates in comparison with reference strains. Next to the nodes the percentage of replicate trees in which the associated taxa clustered together in the bootstrap test with 1,000 replicates are shown. Branch lengths in the tree are scaled in the same units as those of the evolutionary distances (scale bar) used to infer the phylogenetic tree.

16S ribosomal RNA gene sequences had not enough information to discriminate between *B. diazoefficiens* and *B. japonicum*; therefore we used sequences of concatenated *recA*, *atpD*, and *glnII* genes for this purpose (Delamuta et al., 2013). This analysis indicated that five of the *Bradyrhizobium* spp. isolates were related to *B. japonicum*, and the other to *B. diazoefficiens* (Figure 3). By comparing with the DNA-fingerprint of Figure 1, we found that the relatedness of NUJ/N-43 with *B. diazoefficiens* correlated with its position in the DNA-fingerprint in the same branch with the *B. diazoefficiens* strains SEMIA 5080, USDA 122, and USDA 110 with 83% similarity. Meanwhile, CAV/S-14 and CUR/S-25-2 shared the branch with *B. elkanii* USDA 76 and USDA 94 at 74% similarity, but were more related to the *B. diazoefficiens* DNA-fingerprints to which these isolates had 77% similarity. Otherwise, the SNAP-isolates related to *B. japonicum* were more dispersed across the DNA-fingerprint cladogram.

### Stress Tolerance

We tested the 10 rhizobia SNAP-isolates (excluding *P. glycanilyticus*) for their tolerance against four environmental stress conditions that may be relevant at their sites of isolation, namely acidity, aluminum, glyphosate, and heat. All the soils from which the SNAP-isolates were obtained are acid, with soil pH values ranging between 4.8 and 6.4 (Table 1). It is common that such soils also contain high amounts of aluminum in soluble state (Goulding, 2016). In addition, glyphosate is an herbicide widely used in Argentina, where more than 95% of soybean cultivated is transgenic, glyphosate-resistant. Finally, soybean is a summer crop, whereby high soil temperatures are frequent during the crop season.

All the SNAP-isolates tested grew in YMA at pH = 4.8 in the absence or the presence of 50 μM AlCl_3_. Meanwhile, the collection strains *B. japonicum* E109 and *B. diazoefficiens* USDA 110 were able to grow at pH = 4.8, but did not tolerate the presence of AlCl_3_ in the culture medium (Table 2).

Furthermore, we evaluated the resistance of SNAP-isolates to glyphosate according to Moorman et al. (1992). With the exception of *B. japonicum* CAS/N-10 and *R. radiobacter* NUJ/N-44-2, all the SNAP-isolates had more than 70% survival in the presence of glyphosate (Table 2). *B. japonicum* CAS/S-02 and CAV/S-15, and *B. elkanii* CUR/S-25-2 produced equal or more CFU in the presence than in the absence of glyphosate, which may suggest that these isolates might use glyphosate as phosphorous source (Liu et al., 1991). Otherwise, *B. japonicum* E109 survived only 38.6% in the presence of glyphosate.

Depending on the soil surface coverage, soil temperature may reach 40°C between 5 and 10 cm deepness during the summer months in this region while soybean nodulation occurs. Daily, temperature rises from 25 to 28°C at 8:00–9:00 am to reach the peak at 2:00–3:00 pm and then diminishes again during the afternoon. Therefore, we incubated the bacteria with a similar temperature rate of change between 28 and 40°C, and measured the % survival. We observed that all SNAP-isolates, as well as the reference strains *B. diazoefficiens* USDA 110 and *B. japonicum* E109, tolerated this heating-which included a period of 2.5 h at temperatures higher than 35°C-with more than 80% survival (Table 2).

### Motility

We visualized swimming motility in soft agar (0.3%) as dispersion haloes from the point of inoculation. Although all SNAP-isolates were motile, all five *B. japonicum* isolates showed the smaller haloes, while both *B. elkanii* isolates were the most motile (Table 2). The fast-growing *R. radiobacter* isolates were also more motile, but their faster growth rate might also contribute to their dispersion. The *B. diazoefficiens* isolate NUJ/N-43 was quite motile; however, the reference *B. diazoefficiens* USDA 110 had similar motility as *B. japonicum* E109 and the rest of the *B. japonicum* isolates.

### Symbiotic Performance

Nodulation was evaluated through the nodules number and individual nodules dry mass, which were negatively correlated (*r* = -0.56, significant with *p* < 0.05). In this regard, *B. japonicum* CAV/S-15 and SAA/S-48 were outstanding by producing significantly more individual nodules dry mass than the reference strains (Table 3). In addition, *B. diazoefficiens* NUJ/N-43, *B. elkanii* CUR/S-25-2, and *B. japonicum* CAS/N-10 produced similar individual nodules dry mass as the reference strains. At the other extreme, some SNAP-isolates produced nodules with very low individual dry mass. In the case of *B. japonicum* CAS/S-02, *R. radiobacter* NUJ/N-44-1, and *R. radiobacter* NUJ/N-44-2, they produced many nodules but with small size, while *B. elkanii* CAV/S-14 and *B. japonicum* CUR/N-28 produced few nodules of varying size (Table 3). Co-inoculation of *P. glycanilyticus* CUR/S-25-1 with *B. elkanii* CUR/S-25-2, or *R. radiobacter* NUJ/N-44-1 with *R. radiobacter* NUJ/N-44-2, thus recomposing the associations of strains that we isolated from soil or nodules, did not enhance the nodulation of the respective soybean-nodulating isolates inoculated alone (not shown).

**Table 3:** Symbiotic performance of SNAP-isolates. *B. japonicum* E109 and *B. diazoefficiens* USDA 110 were included as reference strains.

Isolate	Nodules	Dry weight			Biomass	Nitrogen content		Nitrogen	Ureides
	per plant	(mg)			Shoot:	(mg g^-1^ dry wt)		Shoot:	(μmol
					Root			Root	g^-1^ leaf
					ratio			ratio	fresh wt)
		Individual	Shoot	Root		Shoot	Root		
		nodule							
*B. japonicum* CAS/N-10	52 C	1.7 BC	533.0 AB	230.6 BC	2.3	27.4 A	14.9 AB	1.8	26.6 A
*B. japonicum* CAS/S-02	98 A	0.8 E	399.4 ABC	215.7 BC	1.8	20.2 B	12.7 DEF	1.6	23.9 AB
*B. elkanii* CAV/S-14	37 C	0.8 DE	348.5 C	207.9 C	1.7	17.0 B	12.8 CDEF	1.3	13.8 C
*B. japonicum* CAV/S-15	41 C	1.7 AB	543.4 A	269.4 AB	2.0	30.1 A	14.1 ABCD	2.1	23.7 AB
*B. japonicum* CUR/N-28	52 BC	0.8 E	348.5 C	207.5 C	1.7	15.2 BC	12.5 DEF	1.2	15.0 BC
*B. elkanii* CUR/S-25-2	33 C	1.5 BC	392.1 ABC	210.4 C	1.9	18.9 B	13.1 BCDE	1.4	21.3 ABC
*B. diazoefficiens* NUJ/N-43	66 B	1.3 CD	545.6 A	248.7 ABC	2.2	26.6 A	15.9 A	1.7	24.1 A
*R. radiobacter* NUJ/N-44-1	73 B	0.7 E	349.5 C	203.4 C	1.7	17.1 B	12.6 DEF	1.4	13.9 C
*R. radiobacter* NUJ/N-44-2	74 B	0.8 DE	454.9 ABC	254.4 ABC	1.8	18.7 B	11.8 EF	1.6	19.0 ABC
*B. japonicum* SAA/S-48	38 C	2.3 A	517.1 ABC	236.0 BC	2.2	28.3 A	15.9 A	1.8	25.5 A
*B. japonicum* E109	49 C	1.4 C	471.0 ABC	205.3 C	2.3	29.0 A	15.9 A	1.8	24.5 A
*B. diazoefficiens* USDA 110	83 AB	1.1 CDE	512.0 ABC	252.1 ABC	2.0	27.0 A	14.8 ABC	1.8	19.8 ABC
Uninoculated control	0	0	367.7 BC	300.7 A	1.2	9.8 C	10.9 F	0.9	13.1 C

*Statistical analysis was performed by ANOVA followed by Tukey test. Values followed by the same letter did not differ with α = 0.01*.

Nitrogen-fixing activity was evaluated by determining the ureide content in leaves, and the total nitrogen accumulated in shoots and roots. When pairwise compared, these parameters were positively correlated in all three combinations (Table 4). Since nitrogen assimilated by the soybean is translocated from roots to shoots as ureides, the concentration of these compounds in leaves is a good indicator of the nitrogen-fixing activity that is taking place in the nodules (Baral et al., 2016). In this regard, most SNAP-isolates were similar to the reference strains, being the ureides contents of only *B. japonicum* CUR/N-28, *B. elkanii* CAV/S-14, and *R. radiobacter* NUJ/N-44-2 lower than the *B. japonicum* E109 reference strain (Table 3). In agreement with the negative *R. radiobacter* NUJ/N-44-1 *nifH* fragment amplification, the N_2_-fixing parameters of plants inoculated with this isolate were similar to uninoculated control.

**Table 4:** Correlation coefficients (*r*) for pairs of symbiotic parameters.

	NDW	SDW	RDW	SN	RN	S/R (B)	S/R (N)
SDW	0,69^∗∗^						
RDW	0,34 NS	0,40 NS					
SN	0,76^∗∗^	0,97^∗∗∗^	-0,03 NS				
RN	0,73^∗∗^	0,84^∗∗∗^	-0,18 NS	0,98^∗∗∗^			
Ureides	0,75^∗∗^	0,88^∗∗∗^	-0,03 NS	0,93^∗∗∗^	0,83^∗∗∗^	0.94^∗∗∗^	0.90^∗∗∗^

^∗∗^*significant with p < 0.01; ^∗∗∗^significant with p < 0.001; NS, non-significant. NDW, nodule dry weight; SDW, shoot dry weight; RDW, root dry weight; SN, shoot total nitrogen; RN, root total nitrogen; S/R (B), shoot:root ratio as total dry biomass; S/R (N), shoot:root ratio as total nitrogen contents*.

Plant growth was evaluated by the shoot and root dry weights, which quantify the total biomass produced by the plants. Since these plants were cultivated with a balanced plant-growth nutrient solution, all the nutrients except nitrogen were supplied at non-limiting levels. While the shoot biomass correlated well with the N_2_-fixation variables, the root biomass showed no correlation (Table 4). However, there were significant differences in root biomass among the plants inoculated with the different SNAP-isolates. Indeed, the uninoculated control had the highest root biomass (*p* < 0.01, Table 3), indicating that variability of root growth in the inoculated plants was not due to external factors such as confinement into the pots.

In our experiments, shoot:root ratios produced by soybean in symbiosis with the different SNAP-isolates measured as total biomass or as total nitrogen were positively correlated between them (*r* = 0.93, significant with *p* < 0.001). Likewise, both shoot:root ratios were positively correlated with the ureide contents in leaves (Table 4). Since ureide contents were obtained from fresh leaf discs while the other magnitudes were obtained from dried shoots, the magnitudes included in the correlations were not mutually influenced.

### Competition for Nodulation

To ensure the conditions for expression of intrinsic competitiveness, we prepared 1:1 admixtures of reference strain and each tested SNAP-isolate from exponential phase cultures, and filled the pots with these admixtures before planting to obtain an even distribution of all bacterial cells (López-García et al., 2002). We observed that none of the SNAP-isolates were more competitive than LP 3018, and only *B. japonicum* CAS/S-02, *B. japonicum* CAS/N-10, and *B. japonicum* SAA/S-48 had similar intrinsic competitiveness as *B. japonicum* LP 3018 (Table 5). These three isolates were grouped together in the DNA-fingerprint cladogram, in a position far away from reference strains (Figure 1).

**Table 5:** Competition for nodulation between each isolate (Sp/Sm-sensitive) and *B. japonicum* LP3018 (Sp/Sm-resistant spontaneous derivative from *B. japonicum* E109).

Isolate	Species	Inoculum ratio	% Sp/Sm-	% Sp/Sm-	Significant
		isolate:LP3018	resistant	sensitive	difference
CAS/N-10	*B. japonicum*	1.0:1.0	70.25	29.75	NS
CAS/S-02	*B. japonicum*	1.1:1.0	60.33	39.67	NS
CAV/S-14-1	*B. elkanii*	1.1:1.0	92.94	7.06	*p* < 0.001
CAV/S-15	*B. japonicum*	1.0:1.0	87.10	12.90	*p* < 0.001
CUR/N-28	*B. japonicum*	1.1:1.0	98.59	1.41	*p* < 0.001
CUR/S-25-2	*B. elkanii*	1.1:1.0	81.41	18.59	*p* < 0.001
NUJ/N-43	*B. diazoefficiens*	1.0:1.0	94.72	5.28	*p* < 0.001
NUJ/N-44-1	*R. radiobacter*	1.1:1.0	94.44	5.56	*p* < 0.001
NUJ/N-44-2	*R. radiobacter*	1.1:1.0	94.44	5.56	*p* < 0.001
SAA/S-48	*B. japonicum*	1.0:1.0	64.58	35.42	NS

*NS, non-significant*.

## Discussion

The 58 SNAP-isolates obtained here with two different methods were grouped in a diversity of DNA-fingerprint genotypes whose distribution correlated with the soil pH of the sampling location. This diversity, however, did not encompass the genotypes of the reference strains that include those most used as inoculants, which were restricted to clade I. Despite the scarce coincidence of DNA-fingerprint genotypes between SNAP-isolates and reference strains, most of the SNAP-isolates belonged to *B. diazoefficiens*, *B. elkanii*, and *B. japonicum*, all used in inoculants. However, we also identified *R. radiobacter* which, to the best of our knowledge, was never used as inoculant in Argentina. This finding suggests that the ability to nodulate soybean might have been acquired by these strains from *Bradyrhizobium* spp. inoculants (Andrews et al., 2018). Horizontal gene transfer from *B. japonicum* to *B. elkanii* was already observed by Minamisawa et al. (2002), and later Silva-Batista et al. (2007) obtained evidence that this phenomenon occurred in soils of Brazil. However, the identity of *nodC* and *nifH* sequences from *R. radiobacter* SNAP-isolates with those from *R. radiobacter* MQ-110s and gx-178, respectively, and their lower relatedness with *Bradyrhizobium* spp. argues against this possibility. Intriguingly, both *R. radiobacter* isolates were observed in close association to each other in a field nodule, although in the laboratory they nodulated when inoculated by separate. In addition, *R. radiobacter* NUJ/N-44-2, from which we could amplify *nifH*, fixed N_2_ at a reasonable level, indicating that the nodules produced by this strain were active. Furthermore, a non-nodulating strain of *P. glycanilyticus* was also found tightly associated to *B. elkanii* in Concepción del Uruguay. Taking into account that the size of our samples is minute in comparison to the whole diversity that probably exists in the soils, the presence of *R. radiobacter* and *P. glycanilyticus* in our small collection suggests that these species are not uncommon members of SNAPs. Previously, the presence of more than one species in a same nodule, including *R. radiobacter* and *P. glycanilyticus*, was reported (Chen et al., 2002; Ampomah and Huss-Danell, 2011; Wu et al., 2011; Martínez-Hidalgo and Hirsch, 2017), indicating that these species might be part of the soil microbiota able to occupy soybean nodules.

Most SNAP-isolates analyzed in more detail manifested tolerance against acidity, aluminum, glyphosate, and heat, which are abiotic stress conditions prevalent in the soybean crop fields in Argentina. By difference to these SNAP-isolates, the inoculant strain was sensitive to aluminum and glyphosate, indicating that the laboratory environment where this inoculant strain is maintained and grown might turn it vulnerable against the selection pressure exerted by the soil environments and the agronomical practices employed. Although we do not know for how long inoculated bacteria may survive the presence of aluminum and glyphosate in the soil (Goulding, 2016; Primost et al., 2017), it is conceivable that these environmental threats may preclude their activity during the root infection process.

The nodulation and N_2_-fixation activities of the SNAP-isolates were diverse, as expected, although there were phenotypes whose N_2_-fixing performances were as high as that of the reference strains. The negative correlation observed between the number and dry mass of individual nodules agrees with the well-established knowledge that, in general, the plant restricts the number of nodules if they are highly active in order to balance the resources allocated to the nodules with the nitrogen nutrient obtained from them (Nishida and Suzaki, 2018). This relationship may be confirmed by the positive correlation observed between the mean individual nodule dry mass with ureide contents in leaves (Table 4). By contrast, the total nodules dry mass per plant showed no correlation at all with ureide contents in leaves (*r* = 0.004) clearly indicating that the nodules with superior dry mass are those responsible for most of the N_2_-fixing activity. The relative ease with which we identified isolates with good symbiotic characteristics indicates that SNAPs may be good sources of strains for better inoculants adapted to their sites of origin. Interestingly, the shoot:root ratio, either of total nitrogen or total biomass, was significantly correlated to shoot nitrogen content and to leaf ureide content. The shoot:root ratio is an indicator of how plants distribute their biomass according to resources availability. Thus, when nutrients are readily available, plant root biomass needs not to increase at the expense of resources that could be used for shoot biomass, contrarily to a situation of scarcity under which more effort should be invested by the root to acquire the limiting nutrients. Our results suggest that, as N_2_-fixation increased with the more efficient SNAP-isolates, more resources could be allocated to shoots growth because extended exploration of the rooting substrate in search for nitrogen became less important. Hence, the shoot:root ratio might be an easy and useful predictor of N_2_-fixing efficacy.

Even considering that the size of our subsample set is small, we consider significant that we could not observe the expected highly competitive phenotypes in any one of the 10 selected SNAP-isolates that we tested against the strain most used as inoculant in Argentina. Indeed, seven were very poor competitors, and only three SNAP-isolates were equally competitive as the reference strain. Interestingly, all these three SNAP-isolates were closely related in the clade VIII of the Box-A1R DNA-fingerprint (Figure 1), which is the most distant from the clade I that contains the collection strains. These results suggest that the lack of highly competitive genotypes observed throughout all SNAP-isolates is not a sampling error. Instead, the prevalence of the observed SNAP genotypes in the soil might be explained by an extrinsic competitive success, in agreement with the notion that external factors, such as the physiological condition or the distribution of the bacterial cells into the soil, are major determinants of the high competitiveness of soil populations (López-García et al., 2001, 2002). Of note, among the seven SNAP-isolates with poor intrinsic competitiveness shown in Table 5, there were two with high N_2_-fixing efficacy, just the contrary than expected (Geetha and Joshi, 2013; Onishchuk et al., 2017). These results are in agreement with the host sanctions hypothesis (Kiers et al., 2003) whereby the host plant promotes premature senescence of nodule cells carrying non-effective rhizobia (Regus et al., 2017), thus maintaining a low proportion of these bacterial genotypes in the population (Daubech et al., 2017).

Previously, we observed both in the laboratory (López-García et al., 2002) and in the field (Althabegoiti et al., 2008; López-García et al., 2009) that seed-inoculated rhizobia are in disadvantage to compete against the soil rhizobia because of the better distribution of the latter in the soil, which is a major determinant of their competitiveness for nodulation (López-García et al., 2002). Although rhizobia are capable of self-propulsion with their flagellar systems at high speeds in liquid media (Quelas et al., 2016a), swimming into the soil is limited by several constraints. Even if we consider a flooded soil, where all its pore space is saturated with water, the paths into the soil pores are tortuous, limiting both bacterial motility and chemoattractants diffusion. Moreover, chemotaxis seems useless in a tortuous environment where long runs would lead the bacteria to constantly hit the soil pore walls (Wolfe and Berg, 1989). When the soil loses its gravitational water, and water contents drops below the field capacity, the remaining soil water is confined to pores of less than ∼30 μm in diameter, which increases bacterial confinement that may limit flagellar rotation. Moreover, water loss leads to increments in the concentration of the soil solution, which augments its viscosity. As a consequence of all these factors, rhizobial net swimming speed into the soil is of the same order as roots elongation rate (Bhuvaneswari et al., 1980; Soby and Bergman, 1983; Barton and Ford, 1995; Turnbull et al., 2001; Covelli et al., 2013). Because the infectible root zone is near the root tip (Bhuvaneswari et al., 1980) rhizobia inoculated on the seeds should “chase” this zone in the elongating roots. This constitutes a disadvantage to the inoculant against the soil rhizobia that are distributed in the soil, which may be “scavenged” by the elongating roots, even without need for the local rhizobia to swim. In fact, Althabegoiti et al. (2011) reported that a non-flagellated mutant of *B. diazoefficiens* was outcompeted by the wild-type for soybean nodulation only when the rooting substrate was flooded but not when it was at field capacity, and in agreement to these observations, in this work we observed no correlation between motility and intrinsic competitiveness, since the most motile SNAP-isolates were among the least competitive (Tables 2, 5), suggesting again that motility in laboratory conditions cannot be extrapolated to soil conditions. Nevertheless, rhizobia bound to a given root region-reached either after self-motility, passive diffusion, or scavenged by the roots-must reach the emerging root hairs to infect (Bhuvaneswari et al., 1980) and this might cause the induction of motility and chemotaxis genes in the rhizosphere (Liu et al., 2017; Salas et al., 2017).

The above results suggest that the inoculant strains, although intrinsically competitive, have low extrinsic competitiveness and impaired persistence in the soil. The inoculant extrinsic competitiveness may be improved by the use of alternative inoculation technologies instead of seed inoculation to ensure better distribution of the rhizobia in the soil (López-García et al., 2009; Lodeiro, 2015), and the low persistence of the inoculant may be avoided with the inclusion in the inoculant formulations of strains with high N_2_-fixation performance and glyphosate and aluminum tolerance selected from the SNAP, which in addition might be experimentally evolved in nodules under local conditions (Martínez et al., 2016). The shoot:root ratio may be a useful indicator in the screening for high N_2_-fixation performance.

## Author Contributions

EI: isolation, genetic characterization, motility, stress tolerance, discussion, and approval of the manuscript. JC: isolation, symbiotic characterization, competition for nodulation, stress tolerance, discussion, and approval of the manuscript. FA: MALDI-TOF MS, discussion, and approval of the manuscript. JP-G: supervision, discussion, and approval of the manuscript. CA-I: supervision, discussion, and approval of the manuscript. AL: direction, funding obtaining, and writing of the manuscript.

## Conflict of Interest Statement

The authors declare that the research was conducted in the absence of any commercial or financial relationships that could be construed as a potential conflict of interest.
