# Supplementary material for: Soybean-Nodulating Strains With Low Intrinsic Competitiveness for Nodulation, Good Symbiotic Performance, and Stress-Tolerance Isolated From Soybean-Cropped Soils in Argentina
Source: Front Microbiol. 2019 May 14;10:1061. doi: 10.3389/fmicb.2019.01061 (PMC6527597; doi:10.3389/fmicb.2019.01061)
Supplement: Supplementary file 1 [file Data_Sheet_1.PDF]

Soybean-nodulating strains with low intrinsic competitiveness for nodulation, good symbiotic performance, and stress-tolerance isolated from soybean-cropped soils in Argentina

**Esteban T. Iturralde, Julieta M. Covelli, Florencia Alvarez,
Julieta Pérez-Giménez, Cesar Arrese-Igor, Aníbal R. Lodeiro**

Supplementary Material

Including:

Table S1: Accession numbers of SNAP-isolates gene sequences used for species determination.

Figure S1: DNA sequences of *nodC* and *nifH* PCR-fragments from *Rhizobium radiobacter*.

Figure S2: Maximum likelihood cladogram for *Rhizobium radiobacter nodC* and *nifH*.

**Table S1** Accession numbers of SNAP-isolates gene sequences used for species determination.		
**Gene**	**Isolate**	**Accession number**
16S rRNA	*Bradyrhizobium diazoefficiens* NUJ/N-43	MK228877
	Bradyrhizobium japonicum CAS/S-02	MK228874
	Bradyrhizobium japonicum CAS/N-10	MK228875
	Bradyrhizobium japonicum CAV/S-15	MK22879
	Bradyrhizobium japonicum CUR/N-28	MK228876
	Bradyrhizobium japonicum SAA/S-48	MK228878
	Bradyrhizobium elkanii CAV/S-14-1	MK228883
	Bradyrhizobium elkanii CUR/S-25-2	MK228880
	Rhizobium radiobacter NUJ/N-44-1	MK228881
	Rhizobium radiobacter NUJ/N-44-2	MK228882
	Paenibacillus glycanilyticus CUR/S-25-1	MK243450
recA	*Bradyrhizobium diazoefficiens* NUJ/N-43	MK246834
	Bradyrhizobium japonicum CAS/S-02	MK246831
	Bradyrhizobium japonicum CAS/N-10	MK246832
	Bradyrhizobium japonicum CAV/S-15	MK246836
	Bradyrhizobium japonicum CUR/N-28	MK246833
	Bradyrhizobium japonicum SAA/S-48	MK246835
atpD	*Bradyrhizobium diazoefficiens* NUJ/N-43	MK246822
	Bradyrhizobium japonicum CAS/S-02	MK246819
	Bradyrhizobium japonicum CAS/N-10	MK246820
	Bradyrhizobium japonicum CAV/S-15	MK246824
	Bradyrhizobium japonicum CUR/N-28	MK246821
	Bradyrhizobium japonicum SAA/S-48	MK246823
glnII	*Bradyrhizobium diazoefficiens* NUJ/N-43	MK246828
	Bradyrhizobium japonicum CAS/S-02	MK246825
	Bradyrhizobium japonicum CAS/N-10	MK246826
	Bradyrhizobium japonicum CAV/S-15	MK246830
	Bradyrhizobium japonicum CUR/N-28	MK246827
	Bradyrhizobium japonicum SAA/S-48	MK246829

NUJ/N-44-1 *nodC*

CTGATGCCAAAGAACGTCGGAAAGCGCAAGGCGCAGATTGTCGCAATACGGGAATCATCGGGAGATTTGGTGCTCAA
CGTTGACTCGGACACGACCATTGCGCCGGACGTAGTCACGAAACTTGCCCTGAAGATGTACAGTCCCGCGGTCGGCG
CGGCGATGGGTCAGTTGACGGCCAGCAACCGCAGCGACACATGGCTGACGCGGTTGATCGACATGGAGTACTGGCT
CGCCTGCAACGAGGAACGAGCAGCACAGGCTCGCTTTGGAGCCGTTATGTGTTGCTGCGGCCCGTGTGCCATGTACC
GGCGGTCCGCACTCCTATTGCTGCTCGATAAATACGAGACGCAACTGTTTCGAGGCAGGCCAAGCGACTTCGGGGAA
GACCGCCACCTCACAATCCTCATGCTGAATGCAGGCTTTCGAACCGAGTACGTTCCGGACGCCATCGCGGCGACGGTC
GTTCCAAACTCGATGGGGGCCTATCTGCGCCAACAACTGCGCTGGGCACGCAGCACGTTTCGCGACACATTGCTCGCG
CTCCGCCTACTGCCGGGCCTTGATCGCTATCTTACGCTGGACGTGATCGGACAGAATCTTGGTCCGCTGCTCCTAGCCC
TCTCGGTCCTGACGGGGCTAGCACAGCTCGCTCTGACGGCCACAGTGCCTTGGTCGACGATCCTGATGATTGCATCTA
TGACAATGGTCCGCTGCGGCGTGGCGGCGTTTCGAGCGCGAGAGCTGCGATTCCTTGGGTTTTCGCTGCACACCCTCC
TCAACGTCGCTCTCCTGCTCCCCCTCAAAGCATATGCGTTGTGCACGTTGAGCAATAGCGACTGGGC

NUJ/N-44-2 *nodC*

AATCGCAACGCCATCATACCTGTACACGATCATTATGCGTGCGACCCGAGGTTCCGCTTTATCCTGATGCCAAAGAACG
TCGAAAAGCGCAAGGCGCAATGTCGCAATACGGGAATCATCGGGAGATTTGGTGCTCAACGTTGACTCGGACACGAC
CATTGCGCCGGACGTAGTCACGAAACTTGCCCTGAAGATGTACAGTCCCGCGGTCGGCGCGGCGATGGGTCAGTTGA
CGGCCAGCAACCGCAGCGACACATGGCTGACGCGGTTGATCGACATGGAGTACTGGCTCGCCTGCAACGAGGAACG
AGCAGCACAGGCTCGCTTTGGAGCCGTTATGTGTTGTGCGGCCCGTGTCCATGTACCGGCGGTCCGCACTCCTATTGC
TGCTCGATAAATACGAGACGCAACTGTTTCGAGGCAGGCCAAGCGACTTCGGGGAAGACCGCCACCTCACAATCTCAT
GCTGAATGCAGGCTTTGAACCGAGTACGTTCCGGACGCCATCGCGGCGACGGTCGTTCCAAACTCGATGGGGGCCTA
TCTGCGCCAACAACTGCGCTGGGCACGCAGCACGTTTCGCGACACATTGCTCGCGCTCCGCCTACTGCCGGGCCTTGA
TCGCTATCTTACGCTGGACGTGATCGGACAGAATCTTGGTCCGCTGCTCCTAGCCCTTCGGTCCTGACGGGGCTAGCA
CAGCTCGCTCTGACGGCCACAGTGCCTTGGTCGACGATCCTGATGATTGCATCTATGACAATGGTCCGCTGCGGCGTG
GCGGCGTTTCGAGCGCGAGAGCTGCGATTCCTTGGGTTTTCGCTGCACACCCTCCTCAACGTCGCTCTCCTGCTCCCCC
TCAAAGCA

NUJ/N-44-2 *nifH*

GGCAAGTCCACCACCACCCAGAACCTCGTCTCCGCCCTGGCCGAAGCCGGTCAGAAGGTGATGATCGTCGGCTGCGA
TCCGAAGGCGGACTCCACCCGTCTGATCCTGCACGCCAAGGCCCAGAACTCGATCATGGAAATGGCCGCCGAAGCCG
GCAGCGTGGAAGACCTCGAGCTCGAAGACGTGCTCAAGGTCGGCTATCGCGACATCAAGTGCGTCGAGTCGGGCGG
CCCTGAGCCGGGCGTTGGCTGCGCTGGCCGCGGTGTGATCACCGCGATCAACTTCCTCGAAGAGGAAGGCGCCTACG
AGGAAGACCTCGACTTCGTGTTCTACGACGTACTCGGCGACGTGGTCTGCGGTGGCTTCGCCATGCCGATCCGCGAG
AACAAGGCGCAGGAGATCTACATCGTCTGCTCCGGCGAAATGATGGCCATGTACGCGGCCAACAACATCGCCAAGGG
CATCGTGAAGTACGCCAACTCCGGCAGCGTGCGTCTGGCAGGCCTGATCTGCAACAGCCGCAACACCGCCCGCGAAG
ACGAACTGATCATGGAACTGGCCCGTCAGCTGGGCACCCAGATGATCCACTTCGTGCCGCGCGACAACGTCGTACAG
CGCGCCGAGATCCGCCGCATGACCGTAGTCGAGTACGACCCGACGGMCAAGCGGCCGACGAGTACCGCCAGCTGGC
GAACAAGATCGTCAACAACCGCAACTTCGTGATTCCGACGCCCATCACCATGGACGAACTCGAAGACAT

Fig. S1 DNA sequences of *nodC* and *nifH* PCR-fragments obtained from the *Rhizobium radiobacter* SNAP-isolates. The *nifH* fragment could not be amplified from NUJ/N-44-1. The highest identities were obtained with *R. radiobacter* (*Agrobacterium tumefaciens*) MQ-110s for both *nodC* and with *R. radiobacter* (*Agrobacterium tumefaciens*) gx-178 for *nifH*.

A

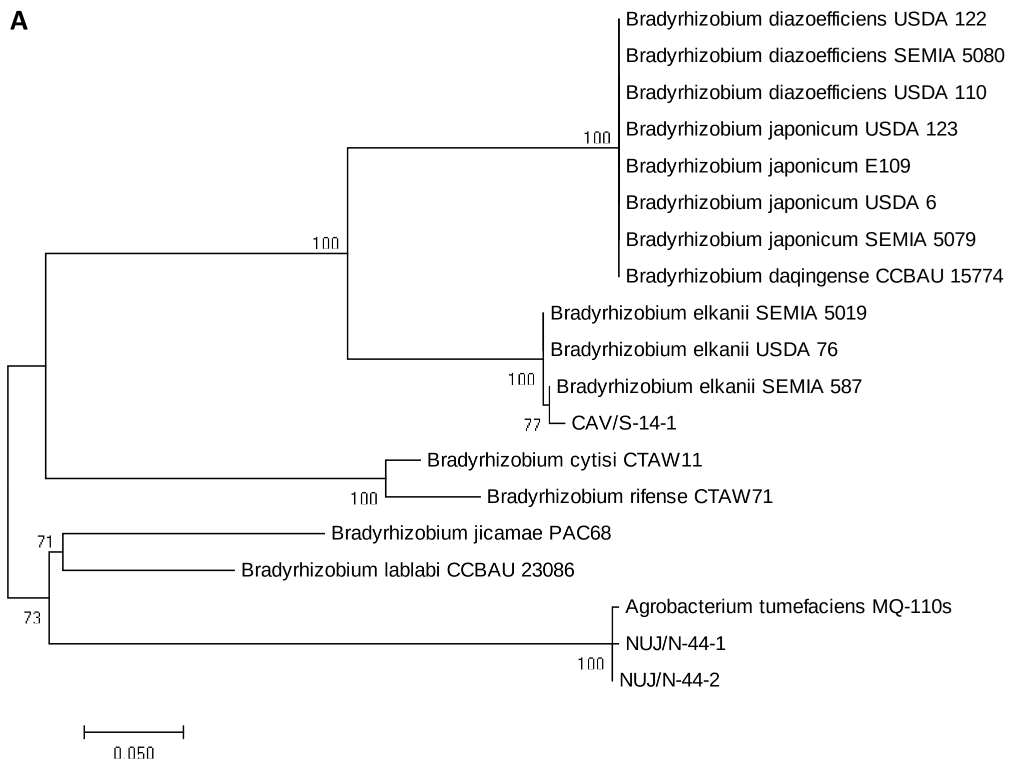

B

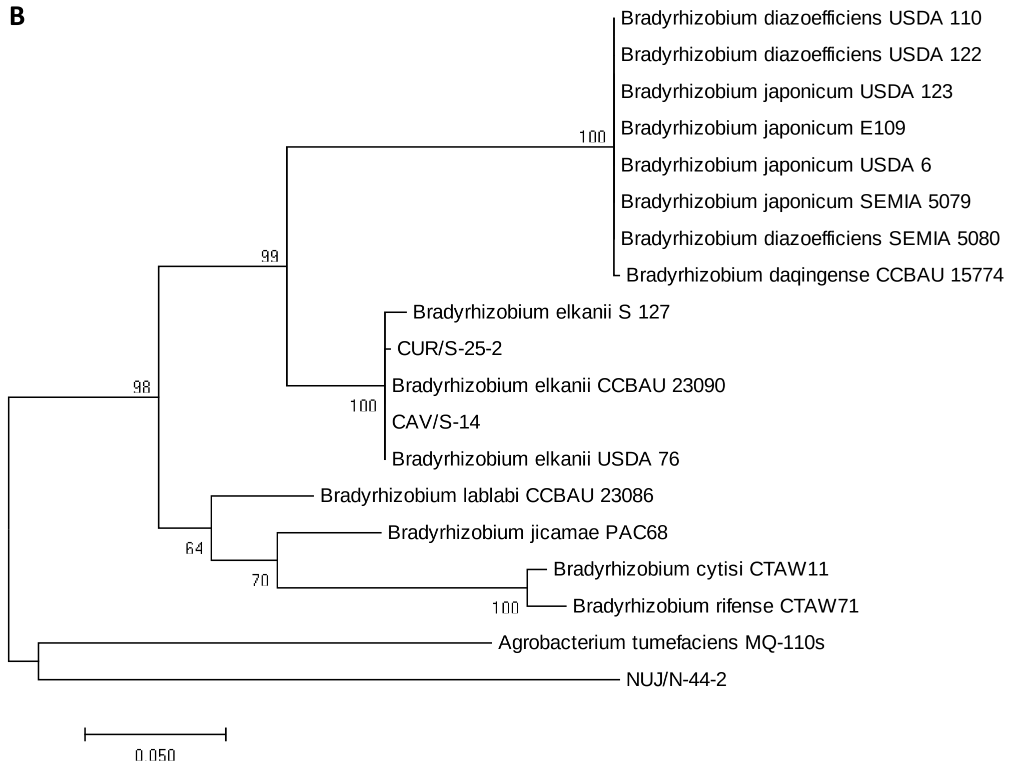

Fig. S2 Maximum likelihood cladogram for *Rhizobium radiobacter* NUJ/N-44-1 and NUJ/N-44-2 *nodC* (**A**) and NUJ/N-44-2 *nifH* (**B**). Next to the nodes the percentage of replicate trees in which the associated taxa clustered together in the bootstrap test with 1000 replicates are shown. Branch lengths in the tree are scaled in the same units as those of the evolutionary distances (scale bar) used to infer the phylogenetic tree.
